# Charge stripes in the graphene-based materials

**DOI:** 10.1038/s41598-023-46157-1

**Published:** 2023-11-02

**Authors:** Petra Grozić, Barbara Keran, Anatoly M. Kadigrobov, Danko Radić

**Affiliations:** 1 Department of Physics, Faculty of Science, University of Zagreb, Bijenička 32, Zagreb, 10000 Croatia; 2 Theoretische Physik III, Ruhr-Universitaet Bochum, Universitätsstraße 150, Bochum, 44801 Germany

**Keywords:** Physics, Condensed-matter physics, Electronic properties and materials

## Abstract

We present an analytical model of the charge density wave instability in graphene sheets within the intercalated graphite CaC_6_ compound. The instability yields the experimentally observed uniaxial charge stripes of periodically modulated electron density, coupled to the softest phonon mode of the superlattice consisting of the Ca atoms intercalated between graphene planes. The Fermi surface of the chemically doped graphene undergoes the novel type of instability driven by the mechanism that gains the condensation energy of the stripe state by the topological reconstruction of the Fermi surface. This mechanism appears to be entirely different from the one based on the Fermi surface nesting, which has been considered a paradigm in the present literature concerning the onset of charge density waves.

## Introduction

The concept of charge density waves (CDW), in broader context also known as charge stripes, has been present in physics since the early prediction of R. Peierls from the 50s of the last century: the homogeneous distribution of electrons in a one-dimensional metal is unstable with respect to their redistribution to a periodically modulated charge density^1^. The condensation energy for the stabilization of such a modulated ground state comes from gapping the Fermi surface by the self-consistently induced periodic potential of the charge density wave. It attains the wave vector precisely determined by the condition to maximize the condensation energy. The experimental realizations appeared in the 80s, in perhaps the most promising real systems with high anisotropy of the chain-like crystal structure, so-called quasi-one-dimensional conductors, such as the blue bronze, Bechgaard salts etc.^2–5^. The high anisotropy of a unit cell leads to also highly anisotropic, open Fermi surface with the unique property of *nesting*—the ability to map parts of the Fermi surface to each other by a unique wave vector. This mimics in the best way the condition of one-dimensionality, required for the Peierls’ mechanism. The perfect match of theory and experiment brought the nesting model to the level of paradigm for the CDW-related physics to the present days. However, later experiments revealed the appearance of charge stripes also in layered, quasi-two-dimensional materials like high-T$_c$ superconducting cuprates^6–8^, intercalated graphite compounds^9,10^, and a number of novel materials such as transition metal dichalcogenides (TMDs)^11^. The common feature of the later systems is the shape of the Fermi surface consisting of closed, more or less isotropic pockets, entirely out of scope of the nesting-based model of the CDW instability. The model based on the topological reconstruction of the Fermi surface of the two-dimensional electron gas with parabolic band was recently proposed^12–14^ giving the qualitative picture of the instability behind such CDWs. The gain in the condensation energy is achieved due to the opening of a pseudo-gap in the electron density of states (DOS), increasing the number of states below the Fermi energy, in turn “pushing” the states in the electron condensate to lower their energy in average, and consequently providing a stable CDW ground state if the electron–phonon coupling is sufficiently large.

In this paper we set our focus to the CDWs in graphene-based compounds, the intarcalated graphite CaC$_6$^15^ in particular. We propose an analytical model which provides a qualitative mechanism leading to the ground state featuring stripes. The model comprises 2D graphene electrons, with Dirac dispersion, coupled to the phonon modes of the superlattice formed of the intercalating Ca atoms. We show that this system becomes unstable with respect to the formation of a uniaxial electron CDW along the graphene planes, due to the mechanism based on the topological reconstruction of the graphene Fermi surface that is critically determined by the chemical doping provided by the Ca atoms. The phonon modes of the intercalant superlattice are much softer than those of graphene, which makes them perfect candidates for the proposed phonon-driven mechanism if coupled strongly enough to graphene electrons. The softest among them undergoes the Kohn anomaly, perfectly reproducing the intercalant lattice distortion as well as direction and periodicity of stripes along the graphene sheets observed in the experiment.

The proposed model explains the following key findings in the experiment performed by Rahnejat et al.^9^: (1) The uniaxial CDW ordering is established below 250 K in the graphene sheets which are chemically doped by electrons from the Ca atoms with 0.2 electrons per carbon atom. Due to the CDW formation, the CaC$_6$ unit cell gets tripled along one of its primitive vectors inclined by 30 degrees with respect to the direction perpendicular to the charge lines. The charge lines are formed along the graphene armchair direction, and are periodically distributed with the distance equal to 4.5 graphene lattice constants along the graphene zig-zag direction. (2) The pseudo-gap appears around the Fermi surface in the CDW-ordered phase. (3) The Ca-superlattice is distorted in a way that every Ca atom along the CDW peak is shifted by $0.06 \pm 0.02$ nm in the same direction, with respect to the other two parallel lines of Ca atoms between the CDW peaks.

## Methods

**Figure 1:**
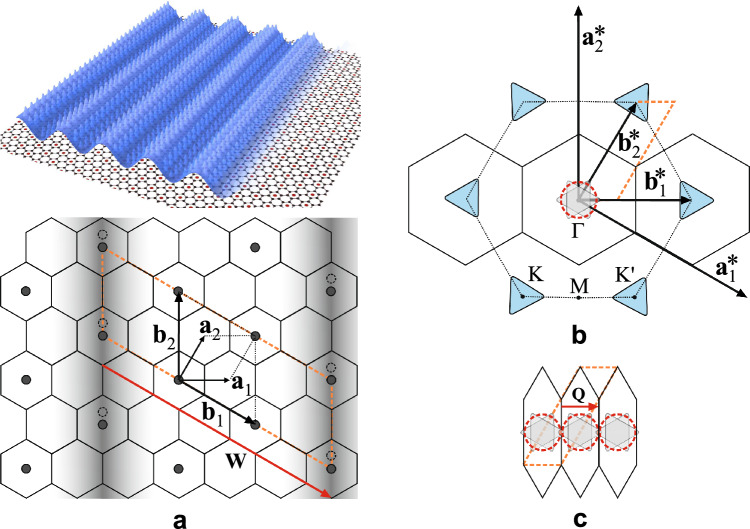
Schematic presentation of one 2D layer in CaC$_6$ system in real (**a**) and reciprocal (**b,c**) space. The unit cell of the CDW-reconstructed system is marked by the dashed orange rhombus in both spaces. (**a**) *Upper panel* In real space, the intercalating Ca atoms (red dots) form the hexagonal superlattice upon the honeycomb graphene lattice (grey). The electron CDW (blue) in the $\pi$-bonds of graphene (chemically doped) spreads periodically along the graphene zig–zag direction. *Lower panel* Carbon atoms form the honeycomb lattice (unit vectors are $\textbf{a}_{1,2}$, $a \equiv \vert \textbf{a}_{1,2} \vert \approx 2.5\, {\text{\AA }}$, the area of the cell is $A_{\text{C}} \approx 5.41\, {\text{\AA }}^2$). Ca atoms (circles) form the hexagonal superlattice (unit vectors are $\textbf{b}_{1,2}$, $b \equiv \vert \textbf{b}_{1,2} \vert = \sqrt{3}a \approx 4.32 \, {\text{\AA }}$, the area of the cell is $A_{\text{CaC6}} \approx 16.16\, {\text{\AA }}^2$). The CDW charge stripes (shaded) are formed along the armchair direction, characterized by the vector $\textbf{W}=3\textbf{b}_1$ that triples the CaC$_6$ cell along $\textbf{b}_{1}$. The Ca-lattice distortion (shift of the Ca atoms under the CDW peaks by $0.06 \pm 0.02$ nm) due to CDW formation is depicted by the dashed circles. (**b**) The carbon Brillouin zone (BZ) is depicted by the dashed hexagon, with standard $\Gamma$, K, K’, M points (unit vectors are $\textbf{a}_{1,2}^*$, $a^* \equiv \vert \textbf{a}_{1,2}^* \vert \approx 2.9\, {\text{\AA }}^{-1}$). The triangles at the K, K’ points represent the Fermi surfaces (FS) of the chemically doped carbon layer^16^. The Ca-superlattice (unit vectors are $\textbf{b}_{1,2}^*$, $b^* \equiv \vert \textbf{b}_{1,2}^* \vert \approx 1.68\, {\text{\AA }}^{-1}$) folds the carbon BZ to a three times smaller CaC$_6$ BZ (solid hexagon). All 6 FSs, from carbon K and K’ points, fall into the $\Gamma$ point (shaded), approximated by a circle of the same area $S_{F0}$ depicted by the dashed red circle. The chemical doping of $\xi \approx 0.2$ electrons per carbon atom is related to the area of the Fermi pocket $S_{F0}=2\pi ^2\xi /A_{\text{CaC6}}\approx 0.244\,{\text{\AA }}^{-2}$, which gives an average Fermi wave number $k_{F0}\approx 0.28\, {\text{\AA }}^{-1}$. (**c**) The CDW potential, with the wave vector $\textbf{Q} \parallel \textbf{b}_{1}^*$ of periodicity $Q=b^*/3 \approx 0.56\, {\text{\AA }}^{-1}$, folds the CaC$_6$ BZ, bringing the FSs into touch (or slight overlap).

The geometry of the problem in real and reciprocal space is presented schematically in Fig. 1. We describe a coupled system of electrons and phonons by the Fröhlich Hamiltonian^17,18^,

1 $$\begin{aligned} H=\sum _{\textbf{k}}\varepsilon (\textbf{k})a^{\dag }_\textbf{k}a_\textbf{k} + \sum _{\textbf{q}}\hbar \omega (\textbf{q})b^{\dag }_\textbf{q}b_\textbf{q} + \frac{1}{\sqrt{A}}\sum _{\textbf{k},\textbf{q}} g_{\textbf{q}} a^{\dag }_{\textbf{k}+\textbf{q}}a_\textbf{k}\left( b^{\dag }_{-\textbf{q}}+b_{\textbf{q}}\right) , \end{aligned}$$

where $a_{\textbf{k}}$ and $b_{\textbf{q}}$ are the standard electron and phonon field operators with the corresponding wave vectors $\textbf{k}$ and $\textbf{q}$ respectively, $\varepsilon (\textbf{k})=\hbar v_F\vert \textbf{k}\vert$ is the Dirac-like electron dispersion with the Fermi velocity $v_F$, $\omega _{\textbf{q}}$ is the phonon frequency and $g_{\textbf{q}}$ is the Fröhlich electron–phonon coupling constant at wave vector ${\textbf{q}}$, *A* is the area of the 2D sample. The formation of the CDW with the wave vector $\textbf{Q}$ implies a static lattice deformation, i.e. a nonvanishing expectation value $\langle b_{\textbf{q}=\textbf{Q}} \rangle$ with corresponding order parameter $\Delta \exp \left( {i \Phi }\right) =2g_{\textbf{Q}} \langle b_\textbf{Q} \rangle /\sqrt{A}$, where $\Delta$ and $\Phi$ are its amplitude and phase. $\textbf{Q}$ and $\Delta$ are to be determined self-consistently by minimization of the total energy of the CDW state, while phase $\Phi$ is not important in the model considering only the ground state, i.e. not considering processes involving some aspects of collective dynamics of the CDW. The Hamiltonian (1) is treated within the mean-field approximation^19^, appearing sufficient to provide a minimal zero-temperature model to reveal the analytical picture explaining the above-counted experimental facts, i.e.

2 $$\begin{aligned} H_{MF}=\sum _\textbf{k}\left[ \varepsilon (\textbf{k})a^{\dag }_\textbf{k}a_\textbf{k} + \Delta e^{i \Phi } a^{\dag }_{\mathbf {k+Q}}a_\textbf{k}+ \Delta e^{-i \Phi } a^{\dag }_{\mathbf {k-Q}}a_\textbf{k}\right] + \frac{A \hbar \omega _{\textbf{Q}}}{2 g_{\textbf{Q}}^{2}} \Delta ^{2}. \end{aligned}$$

**Figure 2:**
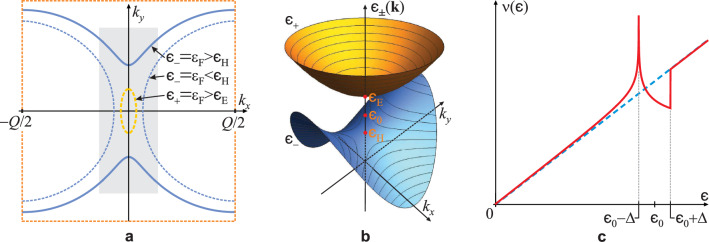
(**a**) The CaC$_6$ Brillouin zone, showing the Fermi surface at energy $\varepsilon _F$ after its reconstruction according to Eq. (3) and initial FS overlap (see Fig. 1c), where $k_x \in (- Q/2,Q/2)$ with origin at the initial band-crossing point. (**b**) The reconstruction region (shaded in (**a**)) with two peculiar points in the $\epsilon _{\pm }(\textbf{k})$ spectrum, a hyperbolic point at energy $\epsilon _H=\epsilon _0-\Delta$ in the lower band $\epsilon _-(\textbf{k})$, and a elliptic point at energy $\epsilon _E=\epsilon _0+\Delta$ in the upper band $\epsilon _+(\textbf{k})$, where $\epsilon _0 = \hbar v_F Q/2$ is the energy of the initial band-crossing point. The contours are cross-sections at constant energy, i.e. $\epsilon _{\pm }(\textbf{k})=\varepsilon _F$. The upper band FS (dashed yellow in (**a**)) appears for Fermi energy $\varepsilon _F > \epsilon _E$. (**c**) DOS of the reconstructed system (red) exhibiting a pseudo-gap appearing between $\epsilon _H$ and $\epsilon _E$, with a diverging van Hove singularity at $\epsilon _H$. The dashed blue line is DOS of the non-reconstructed system, $\nu _0(\epsilon ) = 6\epsilon /(\pi \hbar ^2 v_F^2)$.

The last term in Eq. (2) accounts for the elastic energy of the statically deformed lattice, while the first term is easily diagonalized, yielding the new electron bands

3 $$\begin{aligned} \epsilon _{\pm }(\textbf{k})= \frac{1}{2} \left[ \varepsilon (\textbf{k}-\tfrac{\textbf{Q}}{2})+\varepsilon ( \textbf{k}+\tfrac{\textbf{Q}}{2} ) \pm \sqrt{\left( \varepsilon ( \textbf{k}-\tfrac{\textbf{Q}}{2} )-\varepsilon ( \textbf{k}+\tfrac{\textbf{Q}}{2} ) \right) ^2+4\Delta ^2} \right] , \end{aligned}$$

where we conveniently choose the origin of the reciprocal space at the edge of the new BZ, i.e. the crossing point of the initial electron bands. Due to finite $\Delta$, the degeneracy in the band crossing region is lifted, leading to the reconstruction of the FS. Choosing the coordinate system in which $\hat{\textbf{k}}_x \parallel \textbf{Q}$, it is presented in Fig. 2.

The most fundamental requirement in the reconstruction process of the Fermi surface is the conservation of the number of electrons, i.e. $N_0(\varepsilon _{F0})=N(\varepsilon _{F})$, where $\varepsilon _{F0}$, $\varepsilon _{F}$ are the Fermi energies, and $N_0(\epsilon )$, $N(\epsilon )$ are the numbers of electrons, of the 2D CaC$_6$ system before and after the reconstruction, respectively. The number of electrons is determined by the area of the corresponding (reconstructed or non-reconstructed) Fermi surface $S(\epsilon )$, i.e. $N(\epsilon ) \sim S(\epsilon )/(2\pi )^2$. Taken per unit area in the real space ($A=1$) and assuming the twofold spin degeneracy as well as sixfold FS degeneracy in the $\Gamma$-point of the CaC$_6$ BZ (Fig. 1b), they are

4 $$\begin{aligned} N_0(\epsilon )= & {} 3\epsilon ^2/\bigg (\pi \hbar ^2 v_F^2\bigg ), \nonumber \\ N(\epsilon )= & {} \frac{12}{\pi ^2} \sum _{l=\pm } \int _0^{\frac{Q}{2}}{k_{y,l} (k_x;\epsilon ,Q,\Delta ) \Theta \left[ l \left( K_l(\epsilon ,Q,\Delta ) - k_x \right) \right] \text{d}k_x}. \end{aligned}$$

Here, $k_{y\pm }(k_x;\epsilon ,Q,\Delta )$ should be obtained from Eq. (3) by setting $\epsilon _{\pm }(\textbf{k})=\epsilon$. The Heaviside theta function $\Theta (..)$, where $K_l(\epsilon ,Q,\Delta ) \equiv \tfrac{1}{v_F}\sqrt{(\epsilon -\hbar v_FQ/2)^2-\Delta ^2} \Theta [ l(\epsilon -\hbar v_F Q/2) - \Delta ]$, ensures the real domain of integration over $k_x$ of the $l=\pm$ branch of $k_{y,l}$ depending on the parameters $\epsilon$, *Q*, $\Delta$ (see Fig. 2a). The DOS of the reconstructed system is obtained numerically from Eq. (4), simply as $\nu (\epsilon )=\partial N(\epsilon ) / \partial \epsilon$, and is shown in Fig. 2c, clearly exhibiting a pseudo-gap in the band-crossing region.

Due to the analytical properties of the dispersion (3), it is not possible to obtain $k_{y\pm }(k_x;\epsilon ,Q,\Delta )$ in a closed analytical form. Thus, we use specially developed expansion technique (see Supplemental Material Sect. 1) to obtain analytical results. Namely, adopting the dimensionless variables

5 $$\begin{aligned} \kappa _{x,y} \equiv \frac{k_{x,y}}{Q/2}, \hspace{0.2cm} e_{\pm } \equiv \frac{\epsilon _{\pm }}{\hbar v_F(Q/2)}, \hspace{0.2cm} \delta \equiv \frac{\Delta }{\hbar v_F(Q/2)}, \hspace{0.2cm} e_{F0,F} \equiv \frac{\varepsilon _{F0,F}}{\hbar v_F(Q/2)}, \end{aligned}$$

and assuming $Q \sim 2k_{F0}$, thus constituting $\delta$ and $\Delta e_F \equiv e_F-1$ the small parameters, we divide the $k_x \in (0,Q/2) \rightarrow \kappa _x \in (0,1)$ domain (see Fig. 2a) in two intervals, $(0,\kappa _0)$ and $(\kappa _0,1)$, where $\kappa _0$ is an arbitrary value satisfying the condition $\delta \ll \kappa _0 \ll 1$. Taking into account the smallness of the parameters, we find the expansions of $\kappa _{y\pm }(\kappa _x;e,\delta )$ in the corresponding intervals

6 $$\begin{aligned} \kappa _{y\pm }^<(\kappa _x;e,\delta )= & {} \sqrt{2}\sqrt{e-1 \mp \sqrt{\kappa _x^2+\delta ^2}}, \hspace{0.5cm} \kappa _x \le \kappa _0 \nonumber \\ \kappa _{y\pm }^>(\kappa _x;e,\delta )= & {} \sqrt{e^2-(\kappa _x-1)^2} \pm \frac{1+\sqrt{1+4\kappa _x}}{4\kappa _x^2 \sqrt{2-\kappa _x}} \delta ^2, \hspace{0.5cm} \kappa _x \ge \kappa _0. \end{aligned}$$

The expansion is used in a way that the integral of any function on the $\kappa _x$-interval is divided, e.g. $\int _0^1 f(\kappa _x,\kappa _{y\pm })\text{d}\kappa _x = \int _0^{\kappa _0} f(\kappa _x,\kappa _{y\pm }^<)\text{d}\kappa _x + \int _{\kappa _0}^1 f(\kappa _x,\kappa _{y\pm }^>)\text{d}\kappa _x$, and controlled in a way that, after the expansion of result around $\kappa _0$, the $\kappa _0$-dependent terms should cancel each other (see Supplemental Material Sect. 2).

The condensation energy of the CDW state (per unit area) is the difference of the initial (band) energy, $E_0=\int _0^{\varepsilon _{F0}} \nu _0(\epsilon ) \epsilon \text{d}\epsilon =2\varepsilon _{F0}^3/(\pi \hbar ^2 v_F^2)$, and the final energy of the system which contains contributions of energy of the reconstructed band and the elastic deformation energy of the lattice $\nu _0(\varepsilon _{F0}) \Delta ^2/(2 \lambda )$. The condensation energy normalized to $E_0$ reads

7 $$\begin{aligned} \frac{E_{CDW}}{E_0}= & {} 1 - \frac{3}{2} \left( \frac{Q}{2k_{F0}} \right) ^3 \sum _{l=\pm } 4 \int _0^1 \text{d}\kappa _x \int _0^{\kappa _{y,l}(\kappa _x;e_F,\delta )} e_{l} (\kappa _x,\kappa _y,\delta ) \text{d}\kappa _y \nonumber \\{} & {} - \frac{3}{2\lambda } \left( \frac{\Delta }{\varepsilon _{F0}} \right) ^2, \hspace{1cm} \end{aligned}$$

where $\lambda \equiv \nu _0(\varepsilon _{F0}) g^2/(\hbar \omega _{\textbf{Q}})$ is the dimensionless electron–phonon coupling. In order to stabilize the CDW, the condensation energy has to be positive, with maximum at optimal values of $\varepsilon _F$, *Q* and $\Delta$ obtained through its maximization. In the optimization procedure, the *Q*-dependence of the coupling constant and order parameter is considered to be slow enough to be neglected. The optimization with respect to *Q*, for fixed $\Delta$ yet to be determined, involves only the electron band contribution to the condensation energy. *Q* gets reduced, consequently bringing the occupied parts of the initial electron bands $\varepsilon (\textbf{k}+\textbf{Q}/2)$ and $\varepsilon (\textbf{k}-\textbf{Q}/2)$ into overlap and reconstructing the Fermi surface. Due to the creation of a hyperbolic peculiar point in the lower reconstructed band, at energy $\epsilon _H$ now below the initial Fermi energy $\varepsilon _{F0}>\epsilon _H$, the number of states below $\varepsilon _{F0}$ increases significantly due to formation of the van Hove singularity at $\epsilon _H$ (also evident as the increase of the FS area—see Fig. 2). The Fermi energy of the reconstructed system consequently drops, thus leading to the decrease of the total energy of the reconstructed band. It is evident from Eq. (3) that, for fixed $\Delta$ and initial Fermi energy $\varepsilon _{F0}$ determined by doping, the lower band $\epsilon _-(\textbf{k})$ is filled for any *Q*, while the upper one $\epsilon _+(\textbf{k})$ is filled only for $Q<Q_E \equiv 2(\varepsilon _{F0}-\Delta )/(\hbar v_F)$ which corresponds to energies $\epsilon >\epsilon _E$ (see Fig. 2b). Maximization of the CDW condensation energy for the lower band $\epsilon _-(\textbf{k};\epsilon ,Q,\Delta )$ which is always filled and takes part in the band-reconstruction process, using the Lagrange method with the constraint of conserving the number of particles, shows that the FS overlap increases (reduction of *Q*) until the condensation energy reaches its maximum for $\epsilon =\varepsilon _F=\varepsilon _{F0}$ (see Supplemental Material Sect. 3), *unless* the bottom of the upper band $\epsilon _+(\textbf{k};\epsilon ,Q,\Delta )$ at energy $\epsilon =\epsilon _E$ is reached before that. Filling the upper band decreases the condensation energy and therefore, in that case, the reconstruction process reaches an optimal value of $Q_{opt}=Q_E$, which is the value at which the upper band is formed, leaving $\varepsilon _F<\varepsilon _{F0}$. The conservation of the number of electrons, i.e. $N_0(\varepsilon _{F0})=N(\varepsilon _{F})$, using Eq. (4), with only the lower band taken into account and expanded with respect to the above-mentioned small parameters up to the order $\sim \delta ^2$, yields an equation

8 $$\begin{aligned} \varepsilon _F^2-\varepsilon _{F0}^2 = \sqrt{2} \sqrt{\hbar v_F \tfrac{Q}{2}} \sqrt{\varepsilon _F-\hbar v_F \tfrac{Q}{2}+\Delta }\,\left( \varepsilon _F-\hbar v_F \tfrac{Q}{2}-\Delta \right) -\frac{\alpha }{\pi }\Delta ^2, \end{aligned}$$

where $\alpha = 0.5568$ (see Supplemental Material Sect. 2). The constraint (8) exactly demonstrates that at $\varepsilon _F=\varepsilon _{F0}$, it yields $Q<Q_E$, i.e. the upper band would have been filled. It therefore determines the reconstruction wave vector and Fermi energy of the reconstructed system

9 $$\begin{aligned} Q_{opt} = 2k_{F0} \left[ 1 - \left( \frac{\Delta }{\varepsilon _{F0}} \right) \right] , \hspace{0.5cm} \varepsilon _F = \varepsilon _{F0} \left[ 1 - \frac{\alpha }{2\pi } \left( \frac{\Delta }{\varepsilon _{F0}} \right) ^2 \right] , \end{aligned}$$

(see Supplemental Material Sect. 3). This value of the reconstruction vector is preferably commensurate with the reciprocal lattice constant of the CaC$_6$ structure in the corresponding direction (see Fig. 1), i.e. $b^*=mQ_{opt}$, $m \in \mathbb {N}$. The incommensurability effects will be discussed in the last section. Using values (9) and expanding Eq. (7) in small $\delta$, we obtain

10 $$\begin{aligned} \frac{E_{CDW}}{E_0} = \frac{3}{2} \left[ \frac{1}{\lambda _c} - \frac{1}{\lambda } \right] \left( \frac{\Delta }{\varepsilon _{F0}} \right) ^2 - \frac{3\beta }{2\pi } \left( \frac{\Delta }{\varepsilon _{F0}} \right) ^3, \end{aligned}$$

where $\beta = 5.578$ (see Supplemental Material Sect. 4). Here,

11 $$\begin{aligned} 
\lambda _c \equiv \pi \left[ \frac{\alpha }{3} + \beta \right] ^{-1} \approx 0.54, \end{aligned}$$

appears to be the critical value of the electron–phonon coupling constant, determined by the reconstructed electron band, above which the maximization of condensation energy (10) with respect to the order parameter $\Delta$ possesses a positive maximum. Therefore, for electron–phonon coupling $\lambda > \lambda _c$, the zero-temperature CDW is stabilized with the order parameter $\Delta \approx \tfrac{2\pi }{3\beta } (\lambda _c^{-1}-\lambda ^{-1})\varepsilon _{F0}$.

**Figure 3:**
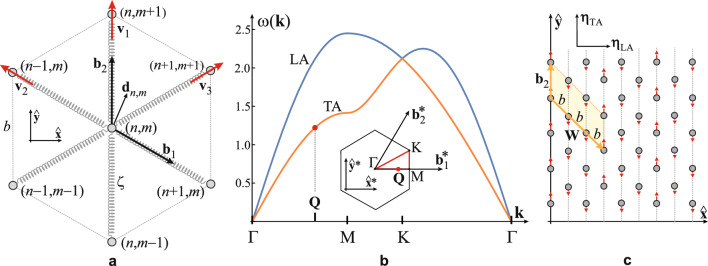
Phonon modes in the 2D hexagonal Ca-lattice (see Fig. 1a). (**a**) Ca atoms of mass *M* in the real space hexagonal structure where (n,m) are atomic positions $\textbf{R}_{nm}=n\textbf{b}_1+m\textbf{b}_2$, *n*, *m* are integers, where $\textbf{b}_1=\tfrac{1}{2}b(\sqrt{3},- 1)$ and $\textbf{b}_2=b(0,1)$ are the unit vectors of the structure in the Cartesian basis $(\hat{\textbf{x}},\hat{\textbf{y}})$. Vibrations of the Ca atoms are described by the first-neighbour harmonic forces, with elastic constant $\zeta$, along three characteristic directions determined by the symmetry, described by unit vectors $\textbf{v}_1=(0,1)$, $\textbf{v}_{2,3}=\tfrac{1}{2}(\mp \sqrt{3},1)$. Displacement of the atom from the equilibrium position $\textbf{R}_{nm}$ is $\textbf{d}_{nm}$. (**b**) Phonon LA and TA in-plane modes $\omega (\textbf{k})$ for the 2D hexagonal lattice in units $\sqrt{\zeta /M}$. The wave vector $\textbf{k}$ is taken along direction between characteristic points in the Brillouin zone (inset) $\Gamma -M-K-\Gamma$. The Ca BZ (see also Fig. 1b) is spanned by reciprocal vectors $\textbf{b}_1^*=\tfrac{4\pi }{\sqrt{3}b}(1,0)$ and $\textbf{b}_2^*=\tfrac{2\pi }{\sqrt{3}b}(1,\sqrt{3})$ in Cartesian reciprocal space $(\hat{\textbf{x}}^*,\hat{\textbf{y}}^*)$. $\textbf{Q}$ is the wave vector of the phonon mode (red dot) related to the CDW. (**c**) Displacements of the Ca atoms (red arrows) $\textbf{d}(\textbf{Q})$ determined by the $\omega _{TA}(\textbf{Q})$ phonon mode: atoms along the CDW peaks are displaced by $\textbf{d}$, while the others are displaced by $-\textbf{d}/2$ along the $\hat{\textbf{y}}$-direction ($\varvec{\eta }_{LA}$ and $\varvec{\eta }_{TA}$ are directions of LA and TA polarization, respectively).

The phonon mode coupled to electron system and responsible for the CDW stabilization is to be found among the in-plane vibrations of the Ca lattice placed between the graphene sheets, being the softest “at hand to nature” to maximize the electron–phonon coupling $\lambda \sim g^2 / \omega _{\textbf{Q}}$, consequently increasing the CDW condensation energy. All graphene vibration modes, as well as the flexural Ca modes, are of much higher frequency thus being neglected in this consideration. The coupling constant *g* will be discussed later. The geometry of the problem is presented in Fig. 3a.

Vibrational modes (phonons) of the 2D Ca lattice are described within the harmonic first-neighbour approximation. The atom displacement from the equilibrium position at $\textbf{R}_{nm}$ is described by the equation

12 $$\begin{aligned} M\frac{\text{d}^2}{\text{d}t^2}\textbf{d}_{nm}=-\zeta \sum _{i=1}^3 \left[ \left( 2\textbf{d}(\textbf{R}_{nm}) - \textbf{d}(\textbf{R}_{nm}+b\textbf{v}_i) - \textbf{d}(\textbf{R}_{nm}-b\textbf{v}_i) \right) \cdot \textbf{v}_i \right] \textbf{v}_i, \hspace{0.5cm} \end{aligned}$$

where *M* is the mass of the atom, $\zeta$ is the linear elasticity constant, *b* is the lattice constant, while $\textbf{v}_i$, $i=1,2,3$, are the unit vectors along three characteristic directions of motion for the hexagonal symmetry (see Fig. 3a). Assuming a solution of Eq. (12) in the standard form, i.e. $\textbf{d}_{nm}=\varvec{\eta } \exp {\text{i}[\textbf{k}\cdot \textbf{R}_{nm} - \omega t]}$, where $\textbf{k}$ is the wave vector, $\omega$ is the frequency and $\varvec{\eta }=(\eta _x,\eta _y)$ is the polarization vector of the phonon mode, Eq. (12) reduces to the secular equation

13 $$\begin{aligned} \left[ \begin{array}{ll} {3\tfrac{\zeta }{M}\left( 1-h_1(\textbf{k})\right) -\omega ^2} &{} \hspace{1cm}{-\sqrt{3}\tfrac{\zeta }{M}h_2(\textbf{k})} \\ \hspace{.5cm}{-\sqrt{3}\tfrac{\zeta }{M}h_2(\textbf{k})} &{} {\tfrac{\zeta }{M}\left( 3-h_1(\textbf{k})-2h_3(\textbf{k})\right) -\omega ^2} \end{array} \right] \left[ \begin{array}{l} {\eta _x}\\ {\eta _y} \end{array} \right] =0, \end{aligned}$$

where $h_1(\textbf{k})\equiv \cos {(\tfrac{\sqrt{3}}{2}k_xb)}\cos {(\tfrac{1}{2}k_yb)}$, $h_2(\textbf{k})\equiv \sin {(\tfrac{\sqrt{3}}{2}k_xb)}\sin {(\tfrac{1}{2}k_yb)}$, $h_3(\textbf{k})\equiv \cos {(k_yb)}$. It yields the dispersion relation of phonon modes

14 $$\begin{aligned} \omega _{\pm }(\textbf{k}) = \sqrt{\tfrac{\zeta }{M}}\left[ 3-2h_1(\textbf{k})-h_3(\textbf{k}) \pm \sqrt{(h_1(\textbf{k})-h_3(\textbf{k}))^2+3h_2(\textbf{k})^2} \right] ^{\frac{1}{2}}, \end{aligned}$$

(see Fig. 3b). The phonon mode related to the CDW has a wave vector $\textbf{Q}=\tfrac{1}{3}\textbf{b}_1^*=\tfrac{4\pi }{3\sqrt{3}b}(1,0)$, which inserted into Eq. (14) yields two frequencies, $\omega _-=\sqrt{3\zeta /2M}$ and $\omega _+=\sqrt{9\zeta /2M}$, and by virtue of Eq. (13), the corresponding polarization unit vectors $\varvec{\eta }_-=(0,1)$ (TA) and $\varvec{\eta }_+=(1,0)$ (LA), respectively. In order to maximize $\lambda \sim g^2/\omega _Q$ we need to compare *g* in the TA and LA directions. The coupling *g* originates from the modification of electron-ion potential due to the ionic shift caused by the phonon. In the spirit of the tight binding approximation (TBA)^20–23^, $g \sim \nabla _{\textbf{R}-\textbf{R}'}t(\textbf{R}-\textbf{R}') \cdot \varvec{\eta }$, where $t(\textbf{R}-\textbf{R}')$ is the TBA transfer integral of electron hopping between sites at positions $\textbf{R}$ (Ca) and $\textbf{R}'$ (carbon) within the first neighbour approximation. From Fig. 1a we see that Ca is centred below the carbon hexagon (thus all $\nabla t$ are equal), but the sum of their projections along the TA is larger than the sum along the LA direction. It is mostly due to the fact that, along the TA, the shift of Ca “strikes” directly into the carbon orbital while it is not the case in the perpendicular LA direction, i.e. $g_{TA} > g_{LA}$. The softer phonon mode between the two, $\omega _-$ (lower frequency, see Fig. 3b), also corresponds to the TA mode with polarization vector $\varvec{\eta }_{TA} \sim \varvec{\eta }_- \parallel \hat{\textbf{y}}$, thus confirming the TA phonon responsible for the CDW. Distortion of the Ca lattice, i.e. displacements of atoms $\textbf{d}_{nm}=\varvec{\eta }_{TA} \cos {[\textbf{Q}\cdot \textbf{R}_{nm}]}$ that corresponds to such polarization and periodicity, is in a way that atoms along the CDW maxima (every 4th column along $\hat{\textbf{y}}$-direction) are shifted by $\varvec{\eta }_{TA}$ while all others are shifted by $-\tfrac{1}{2}\varvec{\eta }_{TA}$ (see Fig. 3c) in terms of amplitude $d=\vert \varvec{\eta }_{TA} \vert$ proportional to the order parameter $\Delta$. That amplitude can be estimated from the mean value of a standard phonon displacement operator $a_0 (b_{\textbf{Q}} + b_{\mathbf {-Q}}^{\dag })$, where $a_0$ is the amplitude of zero-point vibrations, i.e. $d=2\langle b_{\textbf{Q}} \rangle a_0 = a_0 \Delta / g = a_0 \Delta \sqrt{\nu _0(\varepsilon _{F0}) / (\hbar \omega _{\textbf{Q}} \lambda )}$.

## Results and discussion

To compose the results from the above-described methods, we need to put them into context of a real material as much as it is feasible. It is essential to stress that the onset of the CDW originates in the instability of the electron band, through the FS reconstruction which opens the pseudo-gap and decreases the band energy, with the corresponding softened phonon mode that facilitates this mechanism. Therefore, the possibility of establishing a CDW state is crucially limited by the ability of a given system, with its particular geometry of the Brillouin zone, shape and size of the FS, and phonon modes reflecting the symmetry of the lattice, to be compatible with exactly such self-consistent CDW wave vector that relates the FSs in the neighbouring Brillouin zones to touch or very slightly overlap. The CDW wave vector, which determines the overlap for a given FS, must not be too large, neither too short. In the first case there is no overlap, hence no FS reconstruction happens, while in the second case the overlap is too large in the sense that the upper energy band is filled. The CDW condensation energy gets diminished in both cases. It is a known fact that a gap in a band, deeply below the Fermi energy, has no significant influence on the ground state. The softened phonon mode must yield a static deformation of the crystal lattice that is compatible in terms of geometry and periodicity with the CDW. In addition, the electron–phonon coupling to that particular mode must be strong enough, larger than the critical value which is rather high comparing to the weakly coupled Q1D materials^2–5^. That is why most materials do not exhibit the CDW ground state.

**Figure 4:**
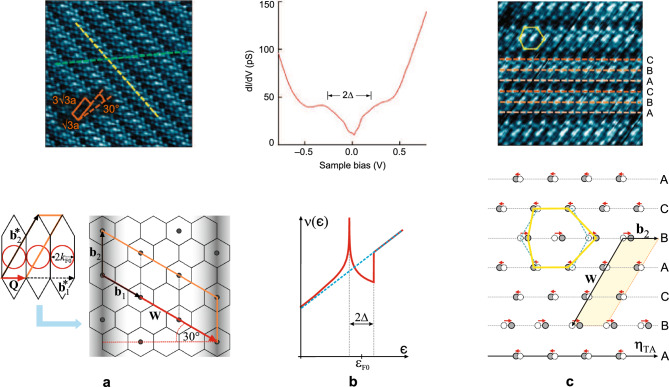
Comparison of experimental results (upper panel, from Ref.^9^, Figs. 2d, 3a and 6a respectively, under Licence by courtesy of Springer Nature) with our theoretical results (lower panel). (**a**) The electron band-driven CDW instability determines the CDW wave vector of length $Q \approx 2k_{F0} = \vert \textbf{b}_1^* \vert /3$ directed along direction of $\textbf{b}_1^*$, which is one of the reciprocal unit vectors $(\textbf{b}_1^*,\textbf{b}_2^*)$ of the CaC$_6$ Brillouin zone that gets shrunken three times (orange rhombus) due to the onset of the CDW. In real space this corresponds to the tripling of the CaC$_6$ unit cell, with unit vectors $(\textbf{b}_1,\textbf{b}_2)$, along the $\textbf{b}_1$ direction (orange rhombus) in the CDW phase, i.e. $\textbf{W}=3\textbf{b}_1$. Charge stripes form peaks (shaded) along the $\textbf{b}_2$ direction. Those peaks appear periodically at a distance equal to 4.5 graphene unit constants $\text{a}$. In the STM experimental picture (upper panel) the stripe peak is along the yellow line, while the orange rhombus is the CDW-extended CaC$_6$ unit cell corresponding to the one in the lower panel where $b=\sqrt{3} \text{a}$. (**b**) Due to the reconstruction of the Fermi surface, the pseudo-gap of width $2\Delta$ is opened in the density of states $\nu (\epsilon )$ approximately around the Fermi energy $\epsilon =\varepsilon _{F0}$, where $\Delta$ is the CDW order parameter. Full red and dashed blue curves depict $\nu (\epsilon )$ after and before the FS reconstruction, respectively. In experiment (upper panel) of measuring the differential conductivity of the STM tunnelling current, which is proportional to LDOS, vs. the bias voltage with respect to the Fermi energy, the pseudo-gap of width $2\Delta$ is observed. (**c**) Static deformation of the initial Ca lattice (dashed circles and blue hexagon) by the “frozen” TA phonon mode due to the CDW: Ca atomic displacements (red arrows) are along the polarization direction $\varvec{\eta }_{TA} \parallel \textbf{b}_2$, while the propagation direction is perpendicular to it. That phonon mode is the softest one at the wave vector $\textbf{Q}$ (see Fig. 3), displacing the Ca atoms at $\textbf{R}_{nm}$ by $\textbf{d}_{nm} \sim \varvec{\eta }_{TA} \cos (\textbf{Q} \cdot \textbf{R}_{nm})$ in a way that chains B (CDW peaks) are displaced by $+d$ and A, C are displaced by $-d/2$ along the $\varvec{\eta }_{TA}$ direction ($d \sim \Delta$). The Ca hexagon in the deformed lattice (full circles) is depicted by yellow contour, corresponding to the experimentally observed STM pattern depicting positions of Ca atoms (upper panel).

The chemical doping of the carbon $\pi$-band with 0.2 electrons per carbon atom, provided by Ca intercalants, creates the Fermi surface with an average diameter $2k_{F0} \approx 0.56\,{\text{\AA }}^{-1}$. That is the characteristic scale of the CDW wave vector $\textbf{Q}$, which is approximately equal to one third of CaC$_6$ reciprocal unit vector $\textbf{b}_1^*$, representing the phonon state on $\Gamma - \text{M}$ line, close to the M-point of the CaC$_6$ Brillouin zone (see Fig. 1b,c). On the other hand, the Ca-intercalated lattice in CaC$_6$ appears to be especially suitable to facilitate the CDW instability for two reasons: (1) Among phonon modes of the Ca lattice, there is a single mode at the wave vector $\textbf{Q}$, exactly compatible with the CDW geometry thus minimizing the “cost” in elastic deformation energy of the lattice (see Fig. 3c). (2) Although there are the ab initio studies on electron–phonon coupling which take into account the intercalating atoms^24–30^, quite a limited number of them is focused to its momentum-resolved value. In contrast to the well-studied problem of superconductivity in CaC$_6$, where the cumulative value or some momentum-averaged value of electron–phonon coupling is relevant, the onset of the CDW instability requires sufficient coupling to the particular phonon mode at wave vector $\textbf{Q}$. Among the above-mentioned studies some recognize the role of *soft* Ca$_{x-y}$ vibrations ($\sim \, 10$ meV) compared to carbon vibrations ($\sim \, 100$ meV)^29,30^ and some indicate the possibility of a significant value of the coupling constant between carbon band and Ca-lattice phonons^26^. The last-mentioned study shows that this electron–phonon coupling is very anisotropic and may attain large value at $\textbf{Q}$ in vicinity of the M-point of the CaC$_6$ Brillouin zone (see Fig. 3b), presumably significantly larger than required critical value of 0.54 predicted by this model. Any coupling larger than critical, from one side directly contributes to the increase of the CDW order parameter $\Delta \sim \lambda - \lambda _c$, but also allows for the fine-tuning of the CDW wave vector. The optimal value of the CDW wave number, $Q_{opt}$, is given by Eq. (9), yielding the value $2k_{F0}$ reduced by the quantity proportional to the order parameter. This value of $Q_{opt}$ is not necessarily commensurate with the unit vectors of the reciprocal lattice. That would lead to the incommensurability effects which decrease the CDW order parameter, reduce the correlation length, create domains and in other ways suppress the CDW ordering. It is known to happen e.g. in cuprates by changing of doping^8,31–33^ or in the TMDs by pressure or intercalation^34–36^. In that respect, with strong enough electron–phonon coupling provided, it is not crucial to provide exactly $Q=Q_{opt}$, the system can self-consistently fine-tune the CDW wave vector close to that value to minimize the negative incommensurability effects. In the CaC$_6$ system it turns out that the (average) diameter of the FS is commensurate by factor 3 with the reciprocal unit vector $\textbf{b}_1^*$ of the reciprocal lattice. Then, even the deviations from the circular shape of the FS used in this model, presumably small with respect to the $\Delta / \hbar v_F$ scale, are not critical for suppressing the CDW ordering (as it is in the “nesting” scenario). It explains the experimentally observed CDW wave vector $\textbf{Q} = \textbf{b}_1^* /3$. That phonon mode gets “frozen” through the Kohn anomaly creating the static deformation of the Ca lattice which stabilizes the CDW. It is a transverse acoustic mode (TA) that displaces the Ca atoms in the direction $\varvec{\eta }_{TA}$ parallel to the CDW peaks, where the displacement is proportional to the order parameter, i.e. $d \sim \langle b_{\textbf{Q}} \rangle \sim \Delta$. Results counted above, directly compared with the experimental findings^9^, are shown in Fig. 4.

In a real material, there is a number of other bands on the Fermi energy^24–27,29,37^, but none of them satisfy the above-mentioned requirements for the CDW instability, therefore they are neglected in the minimal model. Also, in the real material electron band is not the Dirac cone, as it is in the monolayer graphene which is used in this model. CaC$_6$ contains a small gap at the bottom of the conductive band, although the linearity of the band is well-preserved far away from the bottom, around the Fermi energy^26,27,29,37^. Nevertheless, the (neglected) presence of the band-gap at the bottom does not affect our result on condensation energy since it contains the *difference* of initial and reconstructed bands which differ only around the Fermi energy, i.e. the contributions around the bottom of the band cancel each other anyway.

In conclusion, considerable attention has been focused recently on the CDW state observed in materials with closed, rather isotropic Fermi surfaces that entirely exclude the conventional nesting mechanism as its origin. Here, constructing a minimal zero-temperature model based on the topological reconstruction of the Fermi surface from closed pockets to the open sheets, we manage to give a good description of experimentally observed facts in CaC$_6$: (1) the onset of the CDW state with well-predicted size and orientation of its wave vector, (3) the onset of the pseudo-gap around the Fermi energy in the density of states; (3) the exact shape of the in-plane deformation of the Ca superlattice and the onset of that deformation. The presented minimal analytical model, constructed as such mainly for the sake of understanding the underlying mechanisms, in spite of its limitations, explains those key experimental findings thus constituting a solid foundation to quantitatively more rigorous numerical approaches. In particular, the approaches that calculate and consider the momentum-resolved electron–phonon coupling for relevant modes, and also the finite temperature models. In this paper we consider the mean-field commensurate zero-temperature CDW ground state mechanism for which the single phonon-based model is justified. Considering the rather high critical temperature $T_{CDW} \approx 250$ K of the CDW state in CaC$_6$, the more advanced approaches considering the nonadiabatic^38,39^ and anharmonicity effects^40^, such as in the TMDs, might be relevant.

### Supplementary Information

Supplementary Information.

## Data Availability

All data generated or analysed during this study are included in this published article and its supplementary information files.
