# Supplementary material for: Charge stripes in the graphene-based materials
Source: Sci Rep. 2023 Nov 2;13:18931. doi: 10.1038/s41598-023-46157-1 (PMC10622548; doi:10.1038/s41598-023-46157-1)
Supplement: Supplementary file 1 — Supplementary Information. [file 41598_2023_46157_MOESM1_ESM.pdf]

Supplemental Material: Charge Stripes in the Graphene-based Materials

Petra Grozić[1], Barbara Keran[1], Anatoly M. Kadigrobov[1,2] and Danko Radić[1*]

[1]Department of Physics, Faculty of Science, University of Zagreb, Bijenička 32, Zagreb 10000, Croatia.
[2]Theoretische Physik III, Ruhr-Universitaet Bochum, Universitätsstraße 150, Bochum D-44801, Germany.

*Corresponding author(s). E-mail(s): dradic@phy.hr;

1 Reconstructed band structure; expansion

The initial graphene band $\varepsilon(\mathbf{k}) = \hbar v_F |\mathbf{k}|$, where $\mathbf{k}$ is the electron wave vector and v_F is the Fermi velocity, with the origin of the reciprocal space (k_x, k_y) shifted to the edge of the new Brillouin zone imposed by the periodicity vector $\mathbf{Q} = (Q, 0)$

$$\varepsilon_{1,2}(\mathbf{k}) \equiv \varepsilon\left(\mathbf{k} \pm \frac{\mathbf{Q}}{2}\right) = \sqrt{\left(k_x \pm \frac{Q}{2}\right)^2 + k_y^2}, \tag{1}$$

leads to the reconstructed band

$$\epsilon_\pm(\mathbf{k}) = \frac{1}{2}\left[\varepsilon(\mathbf{k}+\tfrac{\mathbf{Q}}{2}) + \varepsilon(\mathbf{k}-\tfrac{\mathbf{Q}}{2}) \pm \sqrt{\left(\varepsilon(\mathbf{k}+\tfrac{\mathbf{Q}}{2}) - \varepsilon(\mathbf{k}-\tfrac{\mathbf{Q}}{2})\right)^2 + 4\Delta^2}\right], \tag{2}$$

after lifting the degeneracy in the origin by a finite gap parameter Δ.

We define the dimensionless variables

$$\kappa_{x,y} \equiv \frac{k_{x,y}}{Q/2}, \quad \xi_{1,2} \equiv \frac{\varepsilon_{1,2}}{\epsilon_0}, \quad e_\pm \equiv \frac{\epsilon_\pm}{\epsilon_0}, \quad \delta \equiv \frac{\Delta}{\epsilon_0}, \quad e_{F0,F} \equiv \frac{\varepsilon_{F0,F}}{\epsilon_0}, \tag{3}$$

where $\epsilon_0 \equiv \hbar v_F Q/2$ is the energy scale, in which the above expressions read

$$\xi_{1,2}(\boldsymbol{\kappa}) = \sqrt{(\kappa_x \pm 1)^2 + \kappa_y^2},$$
$$e_\pm(\boldsymbol{\kappa}, \delta) = \frac{1}{2}\left[\xi_1(\boldsymbol{\kappa}) + \xi_2(\boldsymbol{\kappa}) \pm \sqrt{\left(\xi_1(\boldsymbol{\kappa}) - \xi_2(\boldsymbol{\kappa})\right)^2 + 4\delta^2}\right]. \tag{4}$$

Since only the lower band $e_-(\boldsymbol{\kappa}, \delta) \equiv e(\boldsymbol{\kappa}, \delta)$ contribution takes part in the final result (will be shown later), for simplicity of presentation we present only the corresponding expressions further on. Dividing the $\kappa_x \in (0, 1)$ domain by an arbitrary κ_0 obeying only the condition $\delta \ll \kappa_0 \ll 1$, for the lower band contribution at the energy close to ϵ_0 (see Fig. 2 in the main text and SFig. 1 here), we perform the Taylor expansion within each interval with respect to the small parameter characteristic for that particular interval.

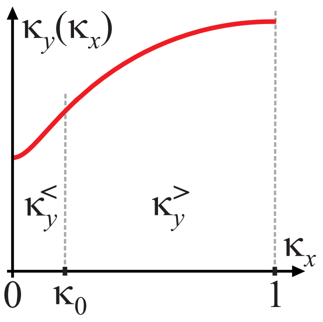

Supp. Fig. 1 Schematic presentation of the division of the $\kappa_x \in (0, 1)$ domain by κ_0 for which $\delta \ll \kappa_0 \ll 1$. $\kappa_y(\kappa_x)$ is approximated by $\kappa_y^<(\kappa_x)$ within $\kappa_x \in (0, \kappa_0)$ and by $\kappa_y^>(\kappa_x)$ within $\kappa_x \in (\kappa_0, 1)$ subintervals.

a) $\kappa_y^<$:
In this limit we use expansion in small $(\kappa_x, \kappa_y) \ll 1$, yielding

$$\xi_{1,2}(\kappa_x, \kappa_y) \approx 1 \mp \kappa_x + \frac{1}{2}\kappa_y^2 + \frac{1}{2}\kappa_x|\boldsymbol{\kappa}|^2,$$
$$e_\pm(\vec{\kappa}, \delta) \approx 1 + \frac{1}{2}\kappa_y^2 \pm \sqrt{\kappa_x^2 + \delta^2},$$

from which we finally obtain

$$\kappa_y^<(\kappa_x, e, \delta) \approx \sqrt{2}\sqrt{e - 1\sqrt{\kappa_x^2 + \delta^2}}. \tag{5}$$

b) $\kappa_y^>$:
In this limit we use expansion in small $\delta \ll (\kappa_x, \kappa_y)$, yielding

$$\kappa_y^{>}(\kappa_x;\ e,\delta) = \sqrt{e^2 - (\kappa_x - 1)^2} + \frac{e^2 + e\sqrt{e^2 + 4\kappa_x}}{4\kappa_x\sqrt{e^2 - (\kappa_x - 1)^2}}\delta^2 + ...,$$

for which, after one more expansion in additional small parameter of the order of δ, i.e. $\Delta e \equiv e - 1 \ll 1$, we get

$$\kappa_y^{>}(\kappa_x;\ e,\delta) = \sqrt{e^2 - (\kappa_x - 1)^2} + \frac{1 + \sqrt{1 + 4\kappa_x}}{4\kappa_x^2\sqrt{2 - \kappa_x}}\delta^2 + \\ + \left(\frac{1 + \frac{1+2\kappa_x}{\sqrt{1+4\kappa_x}}}{2\kappa_x^2\sqrt{2 - \kappa_x^2}} - \frac{1 + \sqrt{1 + 4\kappa_x}}{4\kappa_x(2\kappa_x - \kappa_x^2)^{3/2}} \right)\Delta e\delta^2 + ...,$$

and, keeping accuracy up to $\sim \delta^2$ (later shown to be sufficient), we finally obtain

$$\kappa_y^{>}(\kappa_x;\ e,\delta) \approx \sqrt{e^2 - (\kappa_x - 1)^2} + \frac{1 + \sqrt{1 + 4\kappa_x}}{4\kappa_x^2\sqrt{2 - \kappa_x}}\delta^2. \tag{6}$$

2 Area of the reconstructed Fermi surface; integrals

We define the integral (area of the reconstructed Fermi surface)

$$I = \int_0^1 \kappa_y(\kappa_x;\ e,\delta)d\kappa_x = I^{<} + I^{>}, \tag{7}$$

where

$$I^{<} = \int_0^{\kappa_0} \kappa_y^{<}(\kappa_x;\ e,\delta)d\kappa_x, \\ I^{>} = \int_{\kappa_0}^1 \kappa_y^{>}(\kappa_x;\ e,\delta)d\kappa_x. \tag{8}$$

a) $I^{<}$:

$$I^{<} = \int_0^{\kappa_0} \sqrt{2}\sqrt{\Delta e + \sqrt{\kappa_x^2 + \delta}}\, d\kappa_x \\ \left\{\text{supst. } t \equiv \frac{\kappa_x}{\delta},\ a \equiv \frac{\Delta e}{\delta} > 0,\ r \equiv \frac{\kappa_0}{\delta} \gg 1\right\} \\ = \sqrt{2}\delta^{3/2}\int_0^r \sqrt{a + \sqrt{t^2 + 1}}dt \tag{9}$$

In the Eq. (9), the indefinite integral evaluates to

$$\tilde{I}^{<}(t) = \int \sqrt{a+\sqrt{t^2+1}}dt = \frac{2}{3}\frac{t(2a+\sqrt{t^2+1})}{\sqrt{a+\sqrt{t^2+1}}} + \\ + \frac{2}{3}\sqrt{a+1}\left[a\mathbb{E}\left(\arcsin\frac{\sqrt{a+1}}{\sqrt{a+\sqrt{t^2+1}}}, \frac{a-1}{a+1}\right)\right. \\ \left. -\mathbb{F}\left(\arcsin\frac{\sqrt{a+1}}{\sqrt{a+\sqrt{t^2+1}}}, \frac{a-1}{a+1}\right)\right],$$

where $\mathbb{F}(\phi, m)$ and $\mathbb{E}(\phi, m)$ are the incomplete elliptic integrals of the first and second kind, respectively. Inserting the limits of integration and performing the asymptotic expansion in $r \gg 1$, we obtain

$$\tilde{I}^{<}(r)|_{r\gg 1} \approx \frac{2}{3}r^{3/2} + a\sqrt{r} - \left(\frac{1}{2} - \frac{a^2}{4}\right)\frac{1}{\sqrt{r}} + ..., \\ \tilde{I}^{<}(0) \approx \frac{2}{3}\sqrt{a+1}\left[a\mathbb{E}\left(\frac{\pi}{2}, \frac{a-1}{a+1}\right) - \mathbb{F}\left(\frac{\pi}{2}, \frac{a-1}{a+1}\right)\right] \\ = \frac{2}{3}\sqrt{a+1}\left[a\mathbb{E}\left(\frac{a-1}{a+1}\right) - \mathbb{K}\left(\frac{a-1}{a+1}\right)\right],$$

where $\mathbb{K}(\eta)$ and $\mathbb{E}(\eta)$ are the complete elliptic integrals of the first and second kind, respectively. Using the fact that $\eta \equiv (a-1)/(a+1) \ll 1$ is small, we expand the complete elliptic integrals

$$\mathbb{E}(\eta) \simeq \frac{\pi}{2} - \frac{\pi}{8}\eta - \frac{3\pi}{128}\eta^2 - ..., \\ \mathbb{K}(\eta) \simeq \frac{\pi}{2} + \frac{\pi}{8}\eta + \frac{9\pi}{128}\eta^2 + ...,$$

which,

$$\tilde{I}^{<}(0) = \frac{\pi}{4}\sqrt{a+1}\left[1 - \frac{1}{16}\frac{(a-1)(a+3)}{(a+1)^2}\right] \\ \simeq \frac{\pi}{4}\sqrt{a+1}\,(a-1)\,,$$

where we used $a = \Delta e/\delta$ and $(\Delta e - \delta)/\delta \ll 1$. Using the above, we finally obtain the integral (9), up to the leading terms,

$$I^{<} \approx \sqrt{2}\delta^{3/2}\left[\frac{2}{3}r^{3/2} + a\sqrt{r} - \left(\frac{1}{2} - \frac{a^2}{4}\right)\frac{1}{\sqrt{r}} - \frac{\pi}{4}\sqrt{a+1}(a-1)\right] \\ = \frac{2\sqrt{2}}{3}\kappa_0^{3/2} + \sqrt{2}\Delta e\sqrt{\kappa_0} - \frac{\sqrt{2}}{2}\frac{\delta^2}{\sqrt{\kappa_0}} + \frac{\Delta e^2}{2\sqrt{2}}\frac{1}{\sqrt{\kappa_0}} - \sqrt{2}\frac{\pi}{4}\sqrt{\Delta e+\delta}(\Delta e - \delta). \tag{10}$$

b) $I^>$:
We express the integral as

$$I^> = I_0^> + I_2^>,$$

where

$$I_0^> = \int_{\kappa_0}^{1} \sqrt{e^2 + (\kappa_x - 1)^2} d\kappa_x,$$
$$I_2^> = \delta^2 \int_{\kappa_0}^{1} \frac{1 + \sqrt{1 + 4\kappa_x}}{4\kappa_x^2 \sqrt{2 - \kappa_x}} d\kappa_x. \tag{11}$$

To evaluate the integrals (11), first we take the corresponding indefinite integrals. The first integral is

$$\tilde{I_0}^> (\kappa_x) = \int \sqrt{e^2 - (\kappa_x - 1)^2} d\kappa_x$$
$$= \frac{1}{2}(\kappa_x - 1)\sqrt{e^2 - (\kappa_x - 1)^2} + \frac{1}{2} e^2 \arctan \frac{\kappa_x - 1}{\sqrt{e^2 - (\kappa_x - 1)^2}},$$

in which we insert limits of integration and expand in terms of $\kappa_0 \ll 1$,

$$\tilde{I_0}^> (1) = 0,$$
$$\tilde{I_0}^> (\kappa_0)|_{\kappa_0 \ll 1} \approx -\frac{\pi}{4} e^2 + \frac{\Delta e^2}{2\sqrt{2}\sqrt{\kappa_0}} - \sqrt{2}\sqrt{\kappa_0}\Delta e -$$
$$- \frac{5}{8\sqrt{2}} \sqrt{\kappa_0} \Delta e^2 + \frac{2\sqrt{2}}{3} \kappa_0^{3/2} + \frac{\sqrt{2}}{12} \Delta e \kappa_0^{3/2}.$$

which finally yields the value of the first integral (11) up to the leading terms

$$I_0^> \approx \frac{\pi}{4} e^2 - \frac{\Delta e^2}{2\sqrt{2}\sqrt{\kappa_0}} - \sqrt{2}\sqrt{\kappa_0}\Delta e -$$
$$- \frac{5}{8\sqrt{2}} \sqrt{\kappa_0} \Delta e^2 + \frac{2\sqrt{2}}{3} \kappa_0^{3/2} + \frac{1}{6\sqrt{2}} \kappa_0^{3/2} \Delta e.$$

The second integral is

$$\tilde{I_2}^> (\kappa_x) = \int \frac{1 + \sqrt{1 + 4\kappa_x}}{4\kappa_x^2 \sqrt{2 - \kappa_x}} d\kappa_x =$$
$$= -\frac{1}{4} \frac{\sqrt{2 - \kappa_x}(1 + \sqrt{1 + 4\kappa_x})}{\sqrt{\kappa_x}} +$$
$$+ \frac{3}{4} \left[\mathbb{E} \left(\arcsin \sqrt{1 - \frac{\kappa_x}{2}}, \frac{8}{9} \right) - \mathbb{F} \left(\arcsin \sqrt{1 - \frac{\kappa_x}{2}}, \frac{8}{9} \right) \right],$$

in which we insert limits of integration and expand in terms of $\kappa_0 \ll 1$,

$$\tilde{I_2}^{>}(1) = -\frac{1}{4}\left[1+\sqrt{5}+3\left(\mathbb{F}(\frac{\pi}{4},\frac{8}{9}) - \mathbb{E}(\frac{\pi}{4},\frac{8}{9})\right)\right],$$
$$\tilde{I_2}^{>}(\kappa_0)|_{\kappa_0\ll 1} \approx \frac{3}{4}\left(\mathbb{E}\left(\frac{8}{9}\right) - \mathbb{K}\left(\frac{8}{9}\right)\right) - \frac{1}{\sqrt{2}\sqrt{\kappa_0}} + \frac{5}{4\sqrt{2}}\sqrt{\kappa_0} - $$
$$- \frac{7}{32\sqrt{2}}\kappa_0^{3/2} + ...,$$

which finally yields the value of the second integral (11) up to the leading terms

$$I_2^{>} = \delta^2\left[-\frac{1+\sqrt{5}}{4} - \frac{3}{4}\left(\mathbb{F}\left(\frac{\pi}{4},\frac{8}{9}\right) - \mathbb{E}\left(\frac{\pi}{4},\frac{8}{9}\right) + \mathbb{E}\left(\frac{8}{9}\right) - \mathbb{K}\left(\frac{8}{9}\right)\right) + \right.$$
$$\left. + \frac{1}{\sqrt{2}\sqrt{\kappa_0}} - \frac{5}{4\sqrt{2}}\sqrt{\kappa_0} + \frac{7}{32\sqrt{2}}\kappa_0^{3/2}\right].$$

Collecting all the terms (8), we obtain the integral (7)

$$\begin{aligned} I &\approx I^{<} + I_0^{>} + I_2^{>} \\ &\approx \frac{\pi}{4}e^2 - \sqrt{2}\frac{\pi}{4}\sqrt{\Delta e - \delta}\,(\Delta e - \delta) + \frac{1}{4}\alpha\delta^2, \end{aligned} \tag{12}$$

where

$$\begin{aligned} \alpha &= 3\left(\mathbb{E}\left(\frac{\pi}{4},\frac{8}{9}\right) - \mathbb{F}\left(\frac{\pi}{4},\frac{8}{9}\right) - \mathbb{E}\left(\frac{8}{9}\right) + \mathbb{K}\left(\frac{8}{9}\right)\right) - 1 - \sqrt{5} \\ &\approx 0.5568. \end{aligned} \tag{13}$$

One should note that the final result does not contain contributions containing the arbitrary parameter κ_0, which cancel each other. That also comes as a test of the controlled expansion of the same function in the different regime of parameters.

3 Optimization of the reconstructed band energy with respect to ε_{F0}, ε_F and Q

We start with general expressions for the number of particles (electrons) $N(\varepsilon)$ and the band energy $E_{band}(\varepsilon)$ at energy ε,

$$N(\varepsilon) = \int_0^{\varepsilon} \nu(\varepsilon')d\varepsilon'$$
$$E_{band}(\varepsilon) = \int_o^{\varepsilon} \nu(\varepsilon')\varepsilon' d\varepsilon',$$

in terms of the density of states $\nu(\varepsilon)$. For those we can use an alternative formulation in terms of the 2D equienergy surface $S(\varepsilon)$ (i.e. the Fermi surface for $\varepsilon = \varepsilon_F$),

$$N(\varepsilon) = \sum_{\mathbf{k},s,z_{CaC_6}} 1 = \frac{12}{(2\pi)^2} \int d^2k = \frac{3}{\pi^2} S(\varepsilon)$$
$$E_{band}(\varepsilon) = \int_o^{\varepsilon} \frac{dN}{d\varepsilon'} \varepsilon' d\varepsilon' = \varepsilon N(\varepsilon) - \frac{3}{\pi^2} \int_0^{\varepsilon} S(\varepsilon') d\varepsilon'$$

where $\mathbf{k}$ is the 2D electron momentum and $s = 2$ and $Z_{CaC_6} = 6$ are the spin and CaC_6 degeneracies. The band contribution to the condensation energy is defined as

$$E_{band}^{(c)} \equiv E_{band}^{(0)} - E_{band}^{(r)},$$

where $E_{band}^{(0)}$ is the initial band energy, and $E_{band}^{(r)}$ is the reconstructed band energy. Using the expressions above it reads

$$E_{band}^{(c)} = \varepsilon_{F0} N_0(\varepsilon_{F0}) - \varepsilon_F N_r(\varepsilon_F) + \frac{3}{\pi^2} \left[\int_0^{\varepsilon_F} S_r(\varepsilon) d\varepsilon - \int_0^{\varepsilon_{F0}} S_0(\varepsilon) d\varepsilon, \right], \tag{14}$$

where S_0 and S_r are the initial and reconstructed Fermi surface, N_0 and N_r are the numbers of electrons in the initial and reconstructed system, ε_{F0} and ε_F are the Fermi energies of the initial and reconstructed system. S_r and ε_F depend on Q and Δ, but in this consideration we keep Δ fixed (it is optimized later in the total condensation energy). $E_{band}^{(c)}$ needs to be optimized (i.e. maximized) with respect to ε_F and Q with the condition of conservation of the number of particles in the reconstruction process. In that respect we define the Lagrange function

$$\mathcal{L} = E_{band}^{(c)} - \mu \Delta N,$$

where μ is the Lagrange multiplier and $\Delta N = N_r(\varepsilon_F) - N_0(\varepsilon_{F0})$. Performing the standard optimization procedure, we obtain the following three conditions:

$$\begin{aligned} 1.) \quad 0 &= \frac{\partial \mathcal{L}}{\partial \varepsilon_{F0}} = \nu_0(\varepsilon_{F0})(\varepsilon_{F0} - \mu) \\ 2.) \quad 0 &= \frac{\partial \mathcal{L}}{\partial \varepsilon_F} = \nu(\varepsilon_F)(\varepsilon_{F0} - \varepsilon_F) \\ 3.) \quad 0 &= \frac{\partial \mathcal{L}}{\partial Q} = \int_0^{\varepsilon_F} \frac{\partial S_r(\varepsilon, Q)}{\partial Q} d\varepsilon. \end{aligned} \tag{15}$$

The system (15) shows that the optimal value of the Fermi energy of the reconstructed system is equal to the initial one, i.e. $\varepsilon_F = \varepsilon_{F0}$ as long as there

is $Q = Q_{opt}$ for which $\partial S_r(Q)/\partial Q|_{Q=Q_{opt}} = 0$, i.e. $S_r(Q)$ has a maximum (it is checked by taking the second derivative). It happens only if this maximum is achieved in the lower band *before* the upper band is reached. The presentation of the Fermi surface reconstruction process as Q is varied is shown in SFig. 2, here presented schematically before the calculation for the sake of clarity of presentation. There, it is evident that, taking only the lower band (red contour) into account, as Q is reduced the enclosed area $S(Q)$ is first unchanged, then it starts to increase due to finite gap parameter, and then starts to decrease due to the sole fact that the size of the Brillouin zone $(-Q/2, Q/2)$ is reduced by reduction of Q. This competition apparently creates a maximum of area S_r^{max} at $Q = Q_{opt}$, as it is schematically presented in SFig. 3.

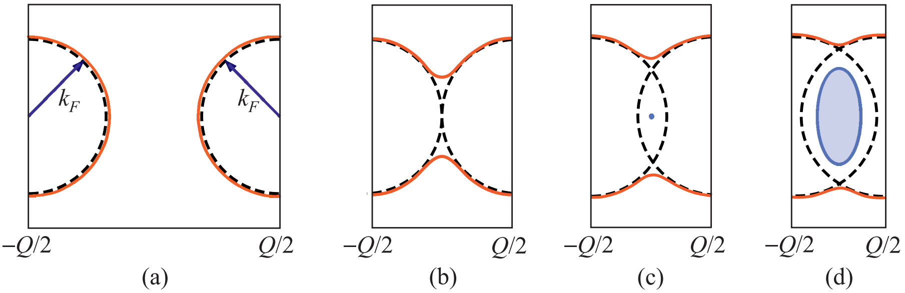

Supp. Fig. 2 Schematic presentation of the 2D Fermi surface reconstruction process at the same Fermi energy (characterized by the Fermi wave number k_F) while Q is varied. Reduction of Q controls the overlap of the initial Fermi surfaces (dashed curve) and size of the new Brillouin zone. The full red curve represents the contour of the reconstructed Fermi surface as Q is reduced, i.e. a) $Q > 2k_F$, b) $Q = 2k_F$, c) $Q < 2k_F$, d) $Q < 2k_F$ and the second (upper) band is formed (blue).

It is important to notice that the scenario described above is valid as long as the maximum $S_r^{max}(Q_{opt})$ appears, by reducing Q, *before* the second (upper) band is formed. If the second band is formed before reaching Q_{opt}, i.e. for $Q > Q_{opt}$, then $E_{band}^{(c)}$ immediately starts to decrease, forming a maximum at that value of Q. Therefore, the reconstruction process goes on by reducing Q until $\varepsilon_F = \varepsilon_{F0}$ where $S_r(\varepsilon_F)$ is maximal *unless* the upper band is reached before. In that case the reconstruction stops at $Q = Q_{opt}$ for which the upper band would be formed and $\varepsilon_F < \varepsilon_{F0}$.

Using the conservation of the number of particles $N_0(\varepsilon_F 0) = N_r(\varepsilon_F)$, i.e. $S_0(\varepsilon_F 0) = S_r(\varepsilon_F)$ in dimensionless variables (3), and integral (12), we get $\pi e_{F0}^2 = 4I$, i.e.

$$\pi e_{F0}^2 = \pi e_F^2 - \sqrt{2}\pi\sqrt{\Delta e_F + \delta}\,(\Delta e_F - \delta) + \alpha\delta^2,$$

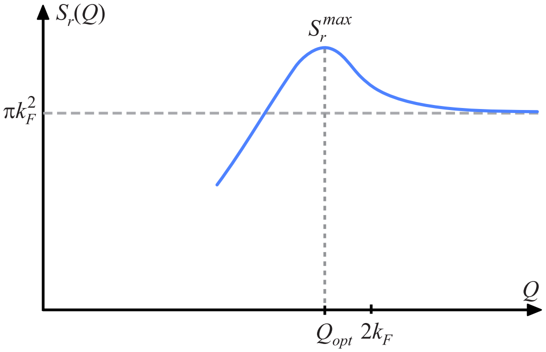

Supp. Fig. 3 Schematic presentation of the change of area of the reconstructed Fermi surface $S_r(Q)$, as Q is varied, of the lower band (only) - see also SFig. 2. For Q significantly larger than $2k_F$, gap parameter has no contribution, yielding the $S_r(Q)$ being equal to πk_F^2. As Q is reduced to $Q \sim 2k_F$, lifting of degeneracy takes place by the (pseudo)gap opening and $S_r(Q)$ increases. By further reduction of Q, $S_r(Q)$ finally starts to decrease because size of the Brillouin zone $(-Q/2, Q/2)$ is decreased by reduction of Q. Therefore, $S_r(Q)$ has a maximum S_r^{max} at $Q = Q_{opt}$.

where $\Delta e_F = e_F - 1$, which, in the original (non-scaled) variables, reads

$$\varepsilon_F^2 - \varepsilon_{F0}^2 = \sqrt{2}\sqrt{\hbar v_F \frac{Q_{opt}}{2}}\sqrt{\varepsilon_F - \hbar v_F \frac{Q_{opt}}{2} + \Delta}\left(\varepsilon_F - \hbar v_F \frac{Q_{opt}}{2} - \Delta\right) - \frac{\alpha}{\pi}\Delta^2 \tag{16}$$

as presented in the main text.

Now, we apply the new scaling (natural scaling, normalization to the initial Fermi energy $\varepsilon_{F0} = \hbar v_F k_{F0}$), i.e. $\tilde{e} = \varepsilon_F/\varepsilon_{F0}$, $q_{opt} = Q_{opt}/(2k_{F0})$, $\tilde{\delta} = \Delta/\varepsilon_{F0}$ ($\tilde{e}_{F0} \equiv 1$), and write the expression (16) in the form

$$\tilde{e}_F^2 - \tilde{e}_{F0}^2 = \sqrt{2}\sqrt{q_{opt}}\sqrt{\tilde{e}_F - q_{opt} + \tilde{\delta}}\left(\tilde{e}_F - q_{opt} - \tilde{\delta}\right) - \frac{\alpha}{\pi}\tilde{\delta}^2. \tag{17}$$

We test if $\tilde{e}_F = \tilde{e}_{F0}$ is achieved before the upper band is reached using Eq. (17). Using perturbation theory for $q_{opt} = q_{opt}^{(0)} + q_{opt}^{(1)}$, $q_{opt}^{(1)} \ll 1$, we get $q_{opt} \approx 1 - \tilde{\delta} - \frac{\alpha}{2\pi}\tilde{\delta}^{3/2}$ which means that the overlap of the initial FS is larger than the critical $q_{opt} = 1 - \tilde{\delta}$ for which the upper band is reached. This means that the reduction of q stops at $q_{opt} = 1 - \tilde{\delta}$. For that value, using perturbation theory in $\tilde{e}_F = \tilde{e}_F^{(0)} + \tilde{e}_F^{(1)}$, $\tilde{e}_F^{(1)} \ll 1$, we obtain $\tilde{e}_F \approx 1 - \frac{\alpha}{2\pi}\tilde{\delta}^2$. It leads to the result $Q_{opt} = 2k_{F0}\left(1 - \frac{\Delta}{\varepsilon_{F0}}\right)$ and $\varepsilon_F = \varepsilon_{F0} - \frac{\alpha}{2\pi}\left(\frac{\Delta}{\varepsilon_{F0}}\right)^2$ presented in the main text.

4 Optimization of the total condensation energy with respect to Δ

4.1 The reconstructed band energy

We calculate the band energy of the reconstructed system, first for an arbitrary Q and Δ, as

$$E_{band}^{(r)} = sZ_{CaC_6}\frac{4}{(2\pi)^2}\int_0^{Q/2} dk_x \int_0^{k_y^{(F)}(k_x,\varepsilon_F,Q,\Delta)} dk_y \epsilon(k_x,k_y,Q,\Delta),$$

where $s = 2$ and $Z_{CaC_6} = 6$ are the spin and CaC_6 degeneracy respectively, $k_y^{(F)}(k_x,\varepsilon_F,Q,\Delta) = k_{y-}(k_x,\epsilon,Q,\Delta)|_{\epsilon=\varepsilon_F}$ and $\epsilon(k_x,k_y,Q,\Delta) = \epsilon_-(k_x,k_y,Q,\Delta)$ from the Eq. (2) (and also the main text). Using the scaling (3), it reads

$$E_{band}^{(r)} = \frac{3}{\pi^2}\hbar v_F \left(\frac{Q}{2}\right)^3 4\int_0^1 d\kappa_x \int_0^{\kappa_y^{(F)}(\kappa_x,e_F,\delta)} d\kappa_y e(\kappa_x,\kappa_y,\delta)$$

where $e(\kappa_x,\kappa_y,\delta) = e_-(\kappa_x,\kappa_y,\delta)$ from Eq. (4). Inserting the optimal value of the wave vector that was found in the previous section, $Q = Q_{opt} = 2k_{F0}(1-\tilde{\delta})$, $\tilde{\delta} \equiv \Delta/\varepsilon_{F0}$, we obtain

$$E_{band}^{(r)} = \frac{3}{\pi^2}\hbar v_F k_{F0}^3 q_{opt}^3 J, \tag{18}$$

where $q_{opt} \equiv 1-\tilde{\delta}$ and

$$J \equiv 4\int_0^1 d\kappa_x \int_0^{\kappa_y^{(F)}(\kappa_x,e_F,\delta)} e(\kappa_x,\kappa_y,\delta) d\kappa_y. \tag{19}$$

To evaluate the integral J, as a function od δ, we develop the following procedure:

$$\frac{dJ}{d\delta} = \frac{\partial J}{\partial e_F}\frac{\partial e_F}{\partial \delta} + \frac{\partial J}{\partial \delta}. \tag{20}$$

Previously, we have found $\varepsilon_F = \varepsilon_{F0}\left(1-\frac{\alpha}{2\pi}\tilde{\delta}^2\right)$, i.e. $e_F = e_{F0}\left(1-\frac{\alpha}{2\pi}\tilde{\delta}^2\right)$, from where we can write $\partial/\partial e_F = \left(1-\frac{\alpha}{2\pi}\tilde{\delta}^2\right)^{-1}\partial/\partial e_{F0}$, i.e. we can change the derivative for the more convenient one. The terms in the expression (20) are calculated below. First, we find

$$\frac{\partial J}{\partial e_{F0}} = 4\int_0^1 d\kappa_x \left(\frac{\partial \kappa_y^{(F)}}{\partial e_{F0}}\right) e(\kappa_x,\kappa_y^{(F)},\delta)$$

$$
\begin{aligned}
&= e_F \frac{\partial}{\partial e_{F0}} 4 \int_0^1 d\kappa_x , \kappa_y^{(F)}(\kappa_x, e_F, \delta) \\
&= e_F \frac{\partial}{\partial e_{F0}} S_r(e_F, \delta),
\end{aligned}
$$

for which, using the conservation of the number of particles $S_r(e_F, \delta) = S_0(e_{F0})$, where $S_0(e_F 0) = \pi e_{F0}^2$, after taking the derivative, we finally get

$$
\frac{\partial J}{\partial e_{F0}} = 2\pi e_F e_{F0}.
$$

Utilizing this result (after changing derivatives) we calculate the first term in Eq. (20)

$$
\begin{aligned}
\frac{\partial J}{\partial e_F} \frac{\partial e_F}{\partial \delta} &= 2\pi e_F e_{F0} \frac{\partial e_{F0}}{\partial \delta} \\
&= 2\pi \left(1 - \frac{\alpha}{2\pi} \tilde{\delta}^2\right) e_{F0}^2 \frac{\partial e_{F0}}{\partial \delta} \\
&= \frac{2\pi}{3} \left(1 - \frac{\alpha}{2\pi} \tilde{\delta}^2\right) \frac{\partial e_{F0}^3}{\partial \delta}.
\end{aligned}
$$

The second term in Eq. (20) is

$$
\begin{aligned}
\frac{\partial J}{\partial \delta} &= 4 \int_0^1 d\kappa_x \left(\frac{\partial \kappa_y^{(F)}}{\partial \delta}\right) e(\kappa_x, \kappa_y^{(F)}, \delta) + 4 \int_0^1 d\kappa_x \int_0^{\kappa_y^{(F)}(\kappa_x, e_F, \delta)} d\kappa_y \frac{\partial}{\partial \delta} e(\kappa_x, \kappa_y, \delta) \\
&= e_F \frac{\partial}{\partial \delta} S_r(e_F, \delta) + 4 \int_0^1 d\kappa_x \int_0^{\kappa_y^{(F)}(\kappa_x, e_F, \delta)} d\kappa_y \frac{-2\delta}{\sqrt{\left(\xi_1(\vec{\kappa}) - \xi_2(\vec{\kappa})\right)^2 + 4\delta^2}}.
\end{aligned}
$$

Again, using the conservation of the number of particles, $S_r(e_F, \delta) = S_0(e_{F0}) = const$, we evaluate the first term of the above expression to zero. The second term is expanded in powers of δ, where a linear term $\sim \delta$ appears from δ in the numerator of the expression (setting it to zero everywhere else). The other contributions (from the denominator and the boundaries of the integral) are of higher order i.e. $\sim \delta^3$. The linear term is sufficient for the final result, therefore we take

$$
\begin{aligned}
\frac{\partial J}{\partial \delta} &\approx \lim_{\delta \to 0} \left[4 \int_0^1 d\kappa_x \int_0^{\kappa_y^{(F)}(\kappa_x, e_F, \delta)} \frac{1}{\sqrt{\left(\xi_1(\vec{\kappa}) - \xi_2(\vec{\kappa})\right)^2 + 4\delta^2}} \right] (-2\delta) \\
&= -2\beta\delta
\end{aligned}
$$

where

$$\beta = 4\int_0^1 d\kappa_x \int_0^{\sqrt{2\kappa_x-\kappa_x^2}} d\kappa_y \frac{1}{\sqrt{(\kappa_x+1)^2+\kappa_y^2}-\sqrt{(\kappa_x-1)^2+\kappa_y^2}}$$
$$= \frac{1}{2}\int_0^1 \frac{d\kappa_x}{\kappa_x}\left[\sqrt{2\kappa_x-\kappa_x^2}\left(1+\sqrt{1+4\kappa_x}\right)+\right.$$
$$\left.+\left(1+\kappa_x\right)^2 \operatorname{arcsinh}\frac{\sqrt{2\kappa_x-\kappa_x^2}}{1+\kappa_x}+\left(1-\kappa_x\right)^2 \operatorname{arcsinh}\frac{\sqrt{2\kappa_x-\kappa_x^2}}{1-\kappa_x}\right]$$
$$= 5.57802 \tag{21}$$

Collecting the terms above, we assemble Eq. (20)

$$\frac{dJ}{d\delta} \approx \frac{2\pi}{3}\left(1-\frac{\alpha}{2\pi}\tilde{\delta}^2\right)\frac{de_{F0}^3}{d\delta} - 2\beta\delta$$

from which we calculate J from Eq. (19) simply by integrating with respect to δ, i.e.

$$J(\delta) \approx \frac{2\pi}{3}\left(1-\frac{\alpha}{2\pi}\tilde{\delta}^2\right)e_{F0}^3 - \beta\delta^2. \tag{22}$$

The energy of the reconstructed band (18) is now easily calculated using $e_{F0} = \varepsilon_{F0}/\left(\hbar v_F Q_{opt}/2\right) = q_{opt}^{-1}$,

$$E_{band}^{(r)} = \frac{3}{\pi^2}\hbar v_F k_{F0}^3 q_{opt}^3\left[\frac{2\pi}{3}\left(1-\frac{\alpha}{2\pi}\tilde{\delta}^2\right)q_{opt}^{-3} - \beta\frac{\tilde{\delta}^2}{q_{opt}^2}\right]$$
$$= E_{band}^{(0)} - \frac{3}{2\pi}E_{band}^{(0)}\left[\left(\frac{\alpha}{3}+\beta\right)\left(\frac{\Delta}{\varepsilon_{F0}}\right)^2 - \beta\left(\frac{\Delta}{\varepsilon_{F0}}\right)^3\right], \tag{23}$$

where $E_{band}^{(0)} = 2\hbar v_F k_{F0}^3/\pi = 2\varepsilon_{F0}^3/(\pi\hbar^2 v_F^2)$ is the energy of the initial, unreconstructed band and the constants α and β are defined in the expressions (13) and (21), respectively.

4.2 "Elastic" energy of the deformed Ca lattice

The last term in the mean-field Hamiltonian of the main text (taken per unit area), usually called "the elastic energy", describes the gain in the energy of the mechanically deformed crystal lattice due to the established CDW ground state coupled to the phonon mode with wave vector $\mathbf{Q}$ and frequency $\omega_{\mathbf{Q}}$. It reads

$$E_{latt} = \frac{\hbar\omega_{\mathbf{Q}}}{2g^2}\Delta^2 = \nu_{F0}\frac{\Delta^2}{2\lambda} = \frac{3}{2}E_{band}^{(0)}\frac{1}{\lambda}\left(\frac{\Delta}{\varepsilon_{F0}}\right)^2, \tag{24}$$

where $\lambda \equiv \nu_{F0} g^2/(\hbar\omega_{\mathbf{Q}})$ is the dimensionless electron-phonon coupling constant for the phonon mode $\omega_{\mathbf{Q}}$ and ν_{F0} is the initial DOS at the Fermi energy. Here we used $\nu_{F0} = 2\varepsilon_{F0}/(\pi\hbar^2 v_F^2)$ and $E_{band}^{(0)} = 2\varepsilon_{F0}^3/(\pi\hbar^2 v_F^2)$ to relate the last two expressions in Eq. (24).

4.3 The condensation energy of the CDW state

The condensation energy of the CDW ground state is defined as a difference between the initial and the final energy of the system, i.e.

$$E_{CDW} = E_{band}^{(0)} - \left(E_{band}^{(r)} + E_{latt}\right).$$

Using expressions (23) and (24), we obtain the result

$$\frac{E_{CDW}}{E_{band}^{(0)}} = \frac{3}{2}\left[\frac{1}{\lambda_c} - \frac{1}{\lambda}\right]\left(\frac{\Delta}{\varepsilon_{F0}}\right)^2 - \frac{3\beta}{2\pi}\left(\frac{\Delta}{\varepsilon_F 0}\right)^3, \tag{25}$$

where

$$\lambda_c \equiv \pi\left[\frac{\alpha}{3} + \beta\right]^{-1} \simeq 0.54, \tag{26}$$

and α and β are defined in expressions (13) and (21), respectively. In order to stabilize the CDW ground state, the condensation energy should be positive for a nonvanishing value of the order parameter. In this case, E_{CDW} has a maximum for $\Delta \neq 0$ if $\lambda > \lambda_c$, i.e. $\partial E_{CDW}/\partial\Delta = 0$ gives the value of the order parameter

$$\Delta = \frac{2\pi}{3\beta}\left[\frac{1}{\lambda_c} - \frac{1}{\lambda}\right]\epsilon_{F0}, \tag{27}$$

for a given electron-phonon coupling λ. For the matter of presentation, we denote the initial band energy $E_{band}^{(0)}$ by E_0 in the main text.
